# Emodin attenuates diabetic kidney disease by inhibiting ferroptosis via upregulating Nrf2 expression

**DOI:** 10.18632/aging.204933

**Published:** 2023-08-07

**Authors:** Jing Ji, Pengyu Tao, Qian Wang, Mengmeng Cui, Mingfeng Cao, Yuzhen Xu

**Affiliations:** 1 Department of Emergency, Shanghai Municipal Hospital of Traditional Chinese Medicine, Shanghai University of Traditional Chinese Medicine, Shanghai 200071, China; 2 Department of Nephrology Seventh People’s Hospital Affiliated to Shanghai University of Traditional Chinese Medicine, Shanghai 200071, China; 3 Department of Central Laboratory, Postdoctoral Workstation, The Affiliated Taian City Central Hospital of Qingdao University, Taian 271000, China; 4 Department of Rehabilitation, The Second Affiliated Hospital of Shandong First Medical University, Taian 271000, China; 5 Department of Endocrinology, The Second Affiliated Hospital of Shandong First Medical University, Taian 271000, China

**Keywords:** DKD, emodin, Nrf2, oxidative stress, ferroptosis

## Abstract

Diabetic kidney disease (DKD) poses a threat to people’s health. The current treatments only provide partial relief of symptoms. Therefore, seeking a promising therapeutic medication for the prevention and control on DKD will benefit patients. Recently, a novel iron-dependent and non-apoptotic regulated mode of cell death, termed as ferroptosis, is expected to offer us a novel insight into the mechanism of DKD. We conducted experiments to investigate the role of ferroptosis in the development of DKD. Iron accumulation, weakened antioxidant capacity and ROS overproduction were observed in the renal tissues of STZ-induced diabetic rats. A persistent high glucose condition contributed to down regulated levels of Glutathione Peroxidase 4 (GPX4) and Solute Carrier Family 7 Member 11 (SLC7A11) which marked the occurrence of ferroptosis. Treatment of Emodin in DKD models could significantly attenuated these changes and reduced renal injury. Besides, NFE2-related factor 2 (Nrf2), an important antioxidant regulator, was inhibited in both *in vivo* and *in vitro* assay, which contributes to Reactive Oxygen Species (ROS) generation that further promoted the expression of ferroptosis related protein. These unwanted effects were offset by the intervention of Emodin. The specific Nrf2 knock out enhanced cell’s sensitivity to ferroptosis by being exposed to high glucose culture, which was improved by treatment of Emodin via restoring activity of Nrf2. In conclusion, our research demonstrated that Emodin exerted renal protection against DKD via inhibiting ferroptosis and restoring Nrf2 mediated antioxidant capacity, which could be employed as a novel therapeutic medication against DKD.

## INTRODUCTION

Diabetic kidney disease (DKD) is referred to a significant microvascular complication of diabetes mellitus (DM) that contributes to substantial mortality and morbidity rates among affected individuals. It serves as a primary pathway leading to the development of end-stage renal disease (ESRD) [1]. Renal biopsy is a golden standard for diagnosing DKD, which is characterized by pathological changes as follows: mesangial expansion, glomerular basement membrane thickening and glomerular sclerosis [2]. The global prevalence of diabetes is estimated to rise to 10.9% by 2045, which make it a public health issue threatening human’s life [3]. Although strict control on hyperglycemia and hypertension are commonly used, the current therapeutic medication fails physician’s expectation for delaying the progression of DKD [4]. Therefore, understanding the mechanism underlying DKD is beneficial for identifying a safe novel drug that can prevent or delay the onset of DKD, which could help address a significant public health burden.

The mechanism of DKD is complex. Recently, a novel type of cell death, termed as ferroptosis, has aroused researchers’ medicinal interest [5]. Unlike necrosis and apoptosis, ferroptosis is a novel form of programmed cell death featuring iron overload and ROS accumulation. An iron balance status in cells is required for normal physiological activities involving ATP production and protein synthesis [6]. However, the excessive accumulation of iron interrupts tissue function, which is detected. Glutathione peroxidase 4 (GPX4) and solute carrier family 7 member 11 (SLC7A11) are two potent anti-oxidant factors regulated by cystine/glutamate, which exert inhibitive effect on ferroptosis [7]. Under diabetic conditions, the dysfunction of System Xc lost its capacity to produce sufficient GPX4 and low expression of SLC7A11 to result in massive production of ROS via Fenton reaction, which initiates the occurrence of ferroptosis [8].

It is clear that pathways mediated reactive oxygen species (ROS) production is associated with ferroptosis, which act a crucial part in the development of DKD [9]. Although antioxidants (vitamin C or E) were employed in the treatment and prevention of DKD by scavenging ROS, the outcome shows less benefits regarding renoprotection [10]. The current treatment approach targeting oxidative stress alone may not represent an optimal strategy for alleviating the severity of DKD. Thus, we assume that reducing ROS induced tissue damage via inhibiting ferroptosis could be employed as an optimal strategy to improve the prognosis in DKD on the basis of close relationship between ferroptosis and ROS.

Nrf2 is an important transcription factor that regulates iron storage. The depletion of Nrf2 is believed to lead to excessive production of iron content deposited in organs and tissues [11]. Nrf2 serves a crucial factor in anti-oxidative stress defense system by regulating the expression of proteins related to oxidation [12]. Therefore, we proposed that enhanced expression Nrf2 is a promising strategy to suppress diabetes-related ferroptosis.

Emodin is the main effective ingredient derived from rhubarb in traditional Chinese medicine. Emodin can improve renal hypertrophy, reduce extracellular matrix accumulation, and inhibit the phenotypic transformation of renal tubular cells, preventing the transition of interstitial fibroblasts from the resting phase to the proliferative phase. Many studies revealed that Emodin is effective in attenuating diabetes related complications by reducing blood glucose level, inhibiting inflammation and restoring energy sensing pathway [13]. But the mechanism underlying these beneficial effects is still in its infancy. Given the central role of ROS induced ferroptosis in the development of DKD, we speculate Emodin may exert its renal protection against DKD via up regulation of Nrf2, an important anti-oxidant factor, inhibiting ferroptosis. Herein, we are going to perform *in vivo* and *in vitro* study to investigate the role of Emodin in regulation of Nrf2 expression to inhibit ferroptosis.

## MATERIALS AND METHODS

### Animal experiments

The diabetic rat model was induced according to the protocol [14]. Healthy male SD rats weighing 200–220 g (*n* = 40) were purchased from Beijing Weitong Lihua Experimental Animal Company. The rats were randomly categorized into four groups (*n* = 10): normal group (NC), STZ induced DM group, STZ induced DM+Fer-1 group (Fer-1) and STZ induced DM + Emodin group (Emodin), and treated for 12 weeks. Experimental protocols were treated on the basis of the Animal Research Ethics Committee of Shanghai College of Traditional Chinese Medicine and approved by the Ethics Committee of the Second Affiliated Hospital of Shandong First Medical University.

### Determination of urinary protein and creatinine

The rats were placed in metabolic cages separately, and urine was collected 24 hours later, and urine volume at week 0 and 12 was recorded. The urine samples of rats in each group were centrifuged at 4°C for 15 minutes, supernatant was collected, urinary albumin and creatinine concentrations were determined by rat urine protein ELISA kit (Shanghai Weiao, China) and rat urine creatinine ELISA kit (Shanghai Weiao, China) respectively. Meanwhile, 24-hour urinary albumin and the ratio of urinary albumin to urinary creatinine (ACR) were calculated.

### Pathological staining

By intraperitoneal injection of 10% chloral hydrate at 0.2 ml/100 g according to their body weight, rats were anesthetized. After the anesthesia took effect, the rats were fixed on the wooden board in supine position, 2/3 area of the rat abdomen was wiped with alcohol cotton ball, and then cut along the midline of the rat abdomen with surgical scissors, and the blood was wiped with gauze while cutting until it reached the lower sternum of the rat. After the abdominal cavity of rats was completely opened, the kidneys of rats were collected. Fresh rat kidney tissue was fixed in 4% paraformaldehyde for more than 24 h. After tissue dehydration, embedding and sectioning, three mice were taken from each group, and HE staining was used to observe glomerular hypertrophy, tubular dilatation, tubular epithelial rupture and tubular dilatation. Masson staining was used to evaluate the level of nephric tubule atrophy and fibrosis. Each rat randomly selected five visual fields, and the area ratio of interstitial fibrosis was analyzed by Image Pro Plus 6.0 software.

### Immunohistochemical staining

Paraffin wax was dewaxed to water, antigen was repaired, 3% BSA or 10% normal rabbit serum was dripped into the circle to cover the kidney tissue evenly, and sealed at room temperature for 30 min. The first antibody was added, carefully shook the blocking solution, dropped the first antibody AT1 (1:1000, Abcam, ab124505), CTGF (1:1000, Abcam, ab6992), Col-I (1:1000, Abcam, ab34710) and Col-III (1:5000, Abcam, ab7778) prepared by PBS on the slices according to a certain proportion, and lay the slices flat in a wet box containing a small amount of water and incubate at 4°C overnight. The second antibody was added (Weiao, Shanghai, China) against the corresponding species to cover the tissue, and incubated at room temperature for 1 h. Semi-quantitative analysis of immunohistochemical integral optical density: under the light microscope of 400 times, 3 rats in each group were randomly selected from 5 visual fields. The Image-Pro Plus 6.0 image was used to analyse the positive integral optical density value per unit area.

### Cell culture

DMEM, a culture medium containing 5.5 mmol/L glucose, 10% FBS (Biological Industries, USA), 100 U/mL penicillin and streptomycin was used for HK-2 cell incubation under suitable culturing condition (37°C, 95% humidity, 5% CO_2_). The HK-2 cells were randomly divided into following groups: (1) HG group, HG + Fer-1 group (1 μm), HG + Emodin group (40 μm), HG + DMSO group (0.1% DMSO), which all received the intervention of culture medium containing 30 mmol/L glucose for 48 h. (2) Si-Nrf2 + Fer-1 group (1 μm), Si-Nrf2+ Emodin group (40 μm), which all received the intervention of culture medium containing 30 mmol/L glucose for 48 h, while Si-Nrf2 + Emodin group (40 μm) cultured in medium containing 30 mmol/L glucose for 48 h. (3) RSL-3 group, RSL-3 + Emodin group which all received the intervention of culture medium containing 30 mmol/L glucose for 48 h [8].

### Determination of iron, MDA, 4-HNE, GSH

The harvested kidney samples were washed with physiological saline. 10 mg of kidney tissues were taken from each sample, and the supernatant was collected by grinding. The supernatant was centrifuged at 4 degrees for 15 minutes, and the values of iron, MDA, 4-HNE and GSH were measured by rat Elisa Kits (Meimian; Jiangsu Industrial Co., Ltd., China) based on the manufacturer’s instructions.

### Transfections

Cells were transfected with 80 nM of siRNA (Genepharma, Shanghai, China) against si-Nrf2 and si-Ctrl, performing with lipofectamine RNAimax (Invitrogen, USA). The procedure was carried out as follows: One tube containing the combination of 8 μL siRNA and 250 μL of Opti-Mem media, another tube containing the combination of 8 μL of lipo 3000 and 250 μL of Opti-Mem media. Then, the two tubes were mixed and incubated at 37°C for 20 minutes. The two tubes were equilibrated at room temperature for 5 minutes. The two tubes were mixed and incubated for 20 minutes at 37°C, then transferred to 6-well cell culture plate to be mixed with 1.5 mL of Opti-MEM serum-free culture medium. Quantitative Real-time PCR was used to confirm the gene silencing induced by siRNA.

### Western blot

The method of western blot was described in our previous papers [15]. The total proteins were extracted the cytoplasmic protein from renal tissues and HK-2 cells, and determined the protein concentration by Bradford method. Electrophoresis was performed on 10% polyacrylamide (SDS-PAGE) gel, and the voltage was 60 V for 30 min and 80 V for 90 min. The protein was separated and transferred to PVDF membrane at room temperature and constant pressure of 100 V for 60 min, and the membrane was washed with TBS buffer for 5 min. The membrane was sealed with 5% skim milk, and incubated with primary antibodies against GPX4 (1:1000; Abcam, ab125066), SLC7A11 (1:1000; Abcam, ab175186), FTH-1 (1:1000; Abcam, ab183781), TFR-1 (1:1000; Abcam, ab285806), Nrf2 (1:1000; Abcam, ab92946), GAPDH (1:5000; Weiao, Shanghai) in the refrigerator at 4 degrees overnight, followed by incubating with secondary antibody. The result was measured by Protein Simple Fluor Chem M, and the gray scale was calculated by IMAGE J. The relative content of target protein is expressed by the ratio of its integral optical density to GAPDH specific band.

### Quantitative real-time PCR

The procedure for quantitative RT-PCR analysis was followed by the manufacturer’s instruction. Total RNA was extracted from renal tissue and HK-2 cells by using trizol reagent (Invitrogen, USA), then transcribed the extracted mRNA to become a cDNA template by using cDNA Synthesis Kit (Thermo Fisher Scientific, USA). Quantitative Real-time PCR experiment was carried out with cDNA as template using SYBR Green master mix (Thermo Fisher Scientific, USA). Ct value was taken (inflection point of amplification dynamic curve), 2−ΔΔCt method was used to calculate the relative expression of the target gene. The quantitative data were analyzed by three independent experiments, and the relative mRNA expression was standardized with GAPDH as internal reference.

### Statistical analysis

The experiments were repeated three times independently. Through the analysis series, we calculate the average value and the standard deviation of the average value. These values were showed as the mean ± standard deviation (S.D.). The results of the quantitative studies were performed by GraphPad Prism 9.4 software. Mann-Whitney test was used to assess the parameters of rats in different groups. If the *P* value < 0.05, the difference was considered statistically significant.

## RESULTS

### Emodin improves the general status of STZ-induced diabetic rats

Diabetes symptoms are referred to “multi-a little more than three", that is polyuria, polydipsia, polyphagia and weight loss. In the normal group, the rats had bright and smooth fur, no hair loss, free movement, flexible response and normal feed and water consumption. However, the status of rats in DM group were manifested by being quiet, less active, rough hair, dull color, as well as more food, more urine output and water consumption compared to normal group, polyuria, shapeless feces, and being quiet and less active. After treatment with Emodin, the above symptoms were improved.

### The ferroptosis induced pathological changes in DM rats

**Figure 1:**
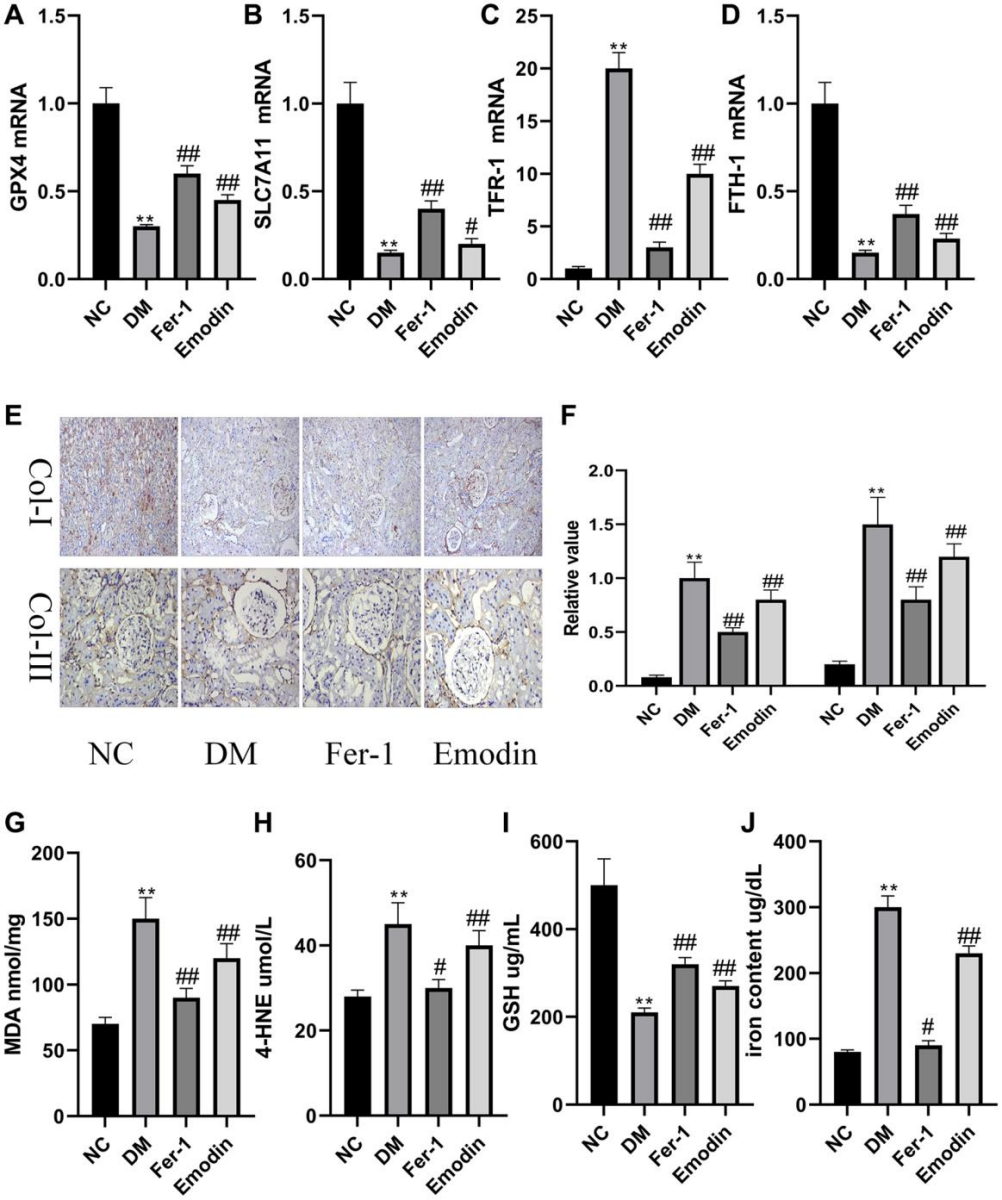
**Ferroptosis induced pathological changes in DM rats.** The mRNA expression of ferroptosis related indicators of renal tissues (**A**–**D**). Immunohistochemical staining (**E**, 200× magnification) and semi-quantification analyses (**F**) of Col-I and Col-III in renal tissues of each group. The concentration of oxidant related indexes in renal tissues was measured (**G**–**J**). ^*^P < 0.05, ^**^P < 0.01 vs. normal group, ^#^*P* < 0.05, ^##^*P* < 0.01 vs. model group.

Both GPX4 and SLC7A11 hold a contributory position in regulating oxidative stress and are often employed as key indicators of ferroptosis. There is considerable evidence that the expression levels of GPX4 and SLC7A11 were prohibited by ROS that promoted the occurrence of ferroptosis. Thus, it is necessary to explore whether Emodin confers health benefits on human body via inhibiting ferroptosis. Our study revealed the decreased mRNA expression levels of GPX4 and SLC7A11 (Figure 1A, 1B) were found in the DM group compared to that of normal group. As shown in Figure 1E, 1F, the immunohistochemical results reflected the renal injuries featuring the enhanced expression of Col-I and Col-III in DM group, which was rescued by Emodin treatment. A prolonged hyperglycemia condition also resulted in the reduction of GSH (Figure 1I), and over generation of MDA (Figure 1G) and 4-HNE (Figure 1H). The above results indicated that the persistent hyperglycemia ambience had destructive influence on the antioxidant function of DM rats, which indicated the initiation of ferroptosis. FTH-1 and TFR-1 are two important genes regulating iron metabolism. We found that the diabetic condition led to decreased expression levels of FTH-1 (Figure 1C) and enhanced TFR-1level (Figure 1D). Our study also revealed that the iron content was obviously increased in renal tissues of rats with STZ induction compared to that of normal group (Figure 1J). Putting together, these data indicated iron overload and loss activity of antioxidant stress system were the leading cause of ferroptosis. It was worth noticing that the intake of Emodin attenuated ferroptosis-related changes in *in vivo* experiments with STZ induction. The mechanism of Emodin inhibiting ferroptosis may be associated with restoring the activation of antioxidant stress system, reducing iron deposition and inhibiting the production of lipid peroxidation products in the Emodin treated group (Figure 1A–1J).

### Emodin improves the renal function of STZ-induced rats

**Figure 2:**
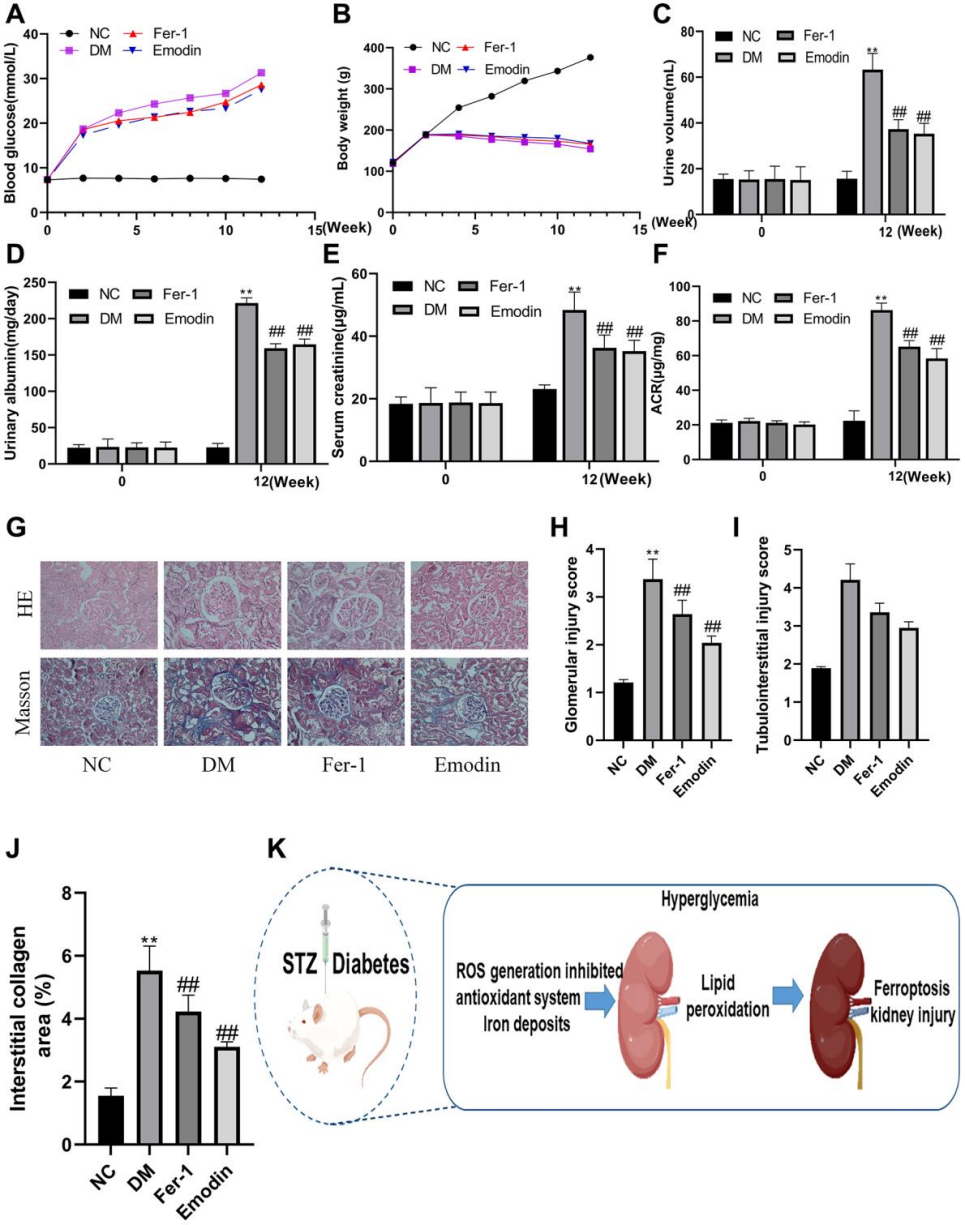
**Emodin attenuated DM related renal injury by inhibiting ferroptosis.** The blood glucose levels and body weight were monitored at different intervention time (**A**, **B**). The measurement of renal function related indicators (**C**–**F**). HE and Masson staining (**G**) 200× magnification) as well as semi-quantitative analysis of glomerular area (**H**–**J**) of renal tissues in each group. The occurrence ferroptosis aggravated the progression of DN (**K**). ^*^P < 0.05, ^**^P < 0.01 vs. normal group, ^#^*P* < 0.05, ^##^*P* < 0.01 vs. model group.

In order to investigate whether Emodin has renal protective effect against DKD, we construct STZ-induced diabetic animal model to explore the protective function of Emodin. The occurrence of proteinuria, rising blood glucose level and increased serum level are considered the hallmark of the development of DKD. As was shown in Figure 2A–2F, the elevated concentration of proteinuria, fasting blood glucose level and serum were observed in STZ-induced diabetic rats group (*P* < 0.05), Both Emodin and Fer-1 treatment significantly attenuated these DM-induced pathological changes. Although the indicators regarding renal impairment remained higher than that of the normal group, they were markedly attenuated in comparison to the untreated model group. The beneficial results were gain from Emodin treatment, including 25% reduction in proteinuria and the serum creatinine decreasing from 48 μg/mL to 35 μg/mL, which indicated an amelioration in renal pathological injuries in diabetic mice.

### Emodin attenuated DM related renal injury by inhibiting ferroptosis

Hyperglycemia is the leading cause of renal injury in DM. Under diabetic condition, a persistent hyperglycemia attacks renal tissue and facilitates the enhanced expression of gene which are responsible for the generation of inflammatory and pro-fibrotic cytokines. Thus, the strict control on hyperglycemia has been proven to be an effective therapy against DKD. Both Masson and HE staining revealed that compared with the NC group, the kidneys of model group exhibited conspicuous renal morphological alterations, such as glomerular changes characterized by the expansion of basement membrane (Figure 2G). The hyperglycemia induced obvious expansion of mesangial leading to aggravation of glomerular injury and tubule interstitial injury that was found in the DM group (Figure 2H–2K). Emodin is an extract derived from Chinese medicine herb plant, which is widely used for the prevention of DM, Fer-1 is a ferroptosis inhibitor. It was worth noticing that both Emodin and Fer-1 treatment exhibited a great potential in attenuating DKD related renal injuries. And these findings indicated the persistent diabetic condition speeding up the deterioration of DKD by promoting ferroptosis, which could be rescued by Emodin treatment.

### High glucose induced ferroptosis in HK-2 cells

**Figure 3:**
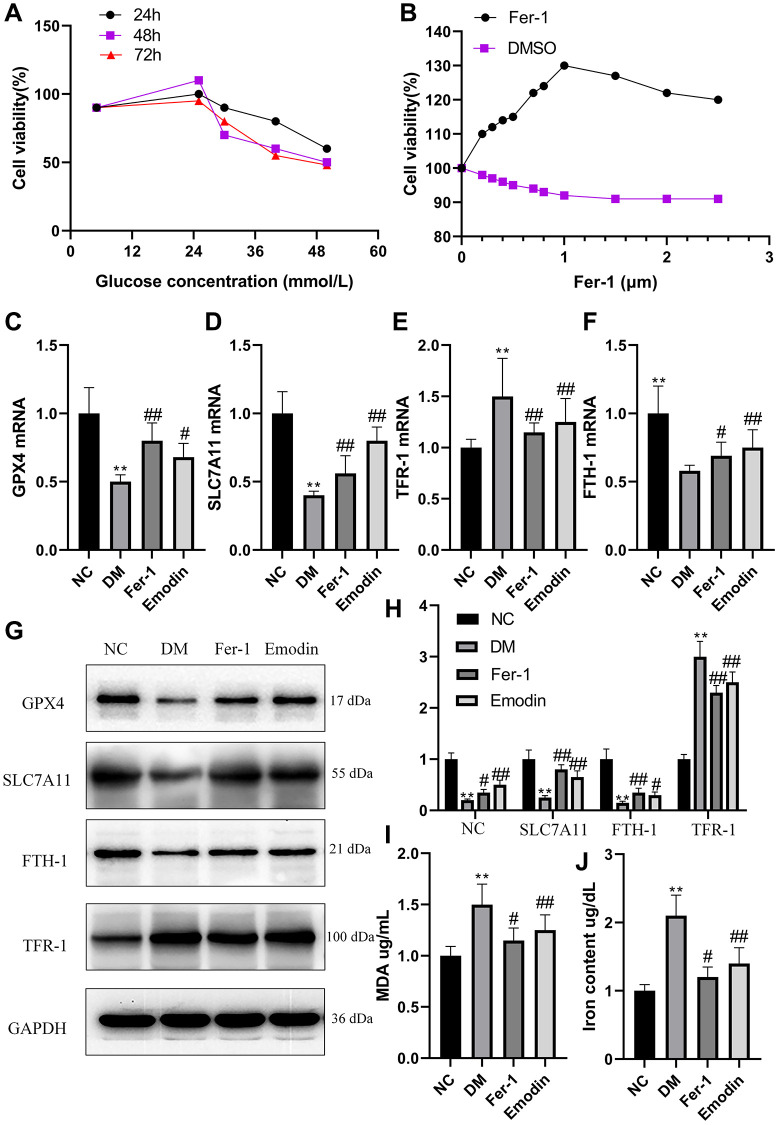
**High glucose induced ferroptosis in HK-2 cells.** The HK-2 cells viability exposure to different concentration of glucose for different time (**A**). The HK-2 cells being exposed to culture medium containing 30 mmol/L glucose and treated with various dose of Fer-1 for a duration of 48 h (**B**). The mRNA expression of ferroptosis related indicators of HK-2 cells (**C**–**F**). The western blotting results (**G**) as well as semi-quantitative analysis (**H**) of ferroptosis related protein expression. The concentration of MDA (**I**) and iron (**J**) of HK-2 cells was measured. ^*^P < 0.05, ^**^P < 0.01 vs. normal group, ^#^*P* < 0.05, ^##^*P* < 0.01 vs. model group.

HK-2 cells were cultured in medium containing glucose concentrations of 5.5, 25, 20, 40 and 50 mmol/L and CCK8 knit was used to detect cell viability at different times (24 h, 48 h and 72 h). The cells viability was slightly higher under the influence of 25 mmol/L compared to those cultured in 5.5 mmol/L. However, the increase in the glucose concentration results in the decline in cell viability (Figure 3A). Interestingly, the supplement of Fer-1 at different concentrations is capable of promoting cell viability, and cell viability reached a peak at 1 μM (Figure 3B). Our study uncovered that HK-2 cells treated with high glucose exhibited decreased mRNA expression levels of GPX4, SLC7A11, and FTH-1 (Figure 3C, 3D, 3F), and conversely, increased mRNA expression levels of TFR-1 (Figure 3E) in comparison with Ctrl group. The WB results also reflected an identical change in the ferroptosis related protein expression levels, including GPX4, SLC7A11, FTH-1 and TFR-1 (Figure 3G, 3H). The high glucose treated group generated massive amount of MDA and iron content compared with ctrl group (Figure 3I, 3J).

### The deletion of Nrf2 induced ferroptosis in HK-2 cells

**Figure 4:**
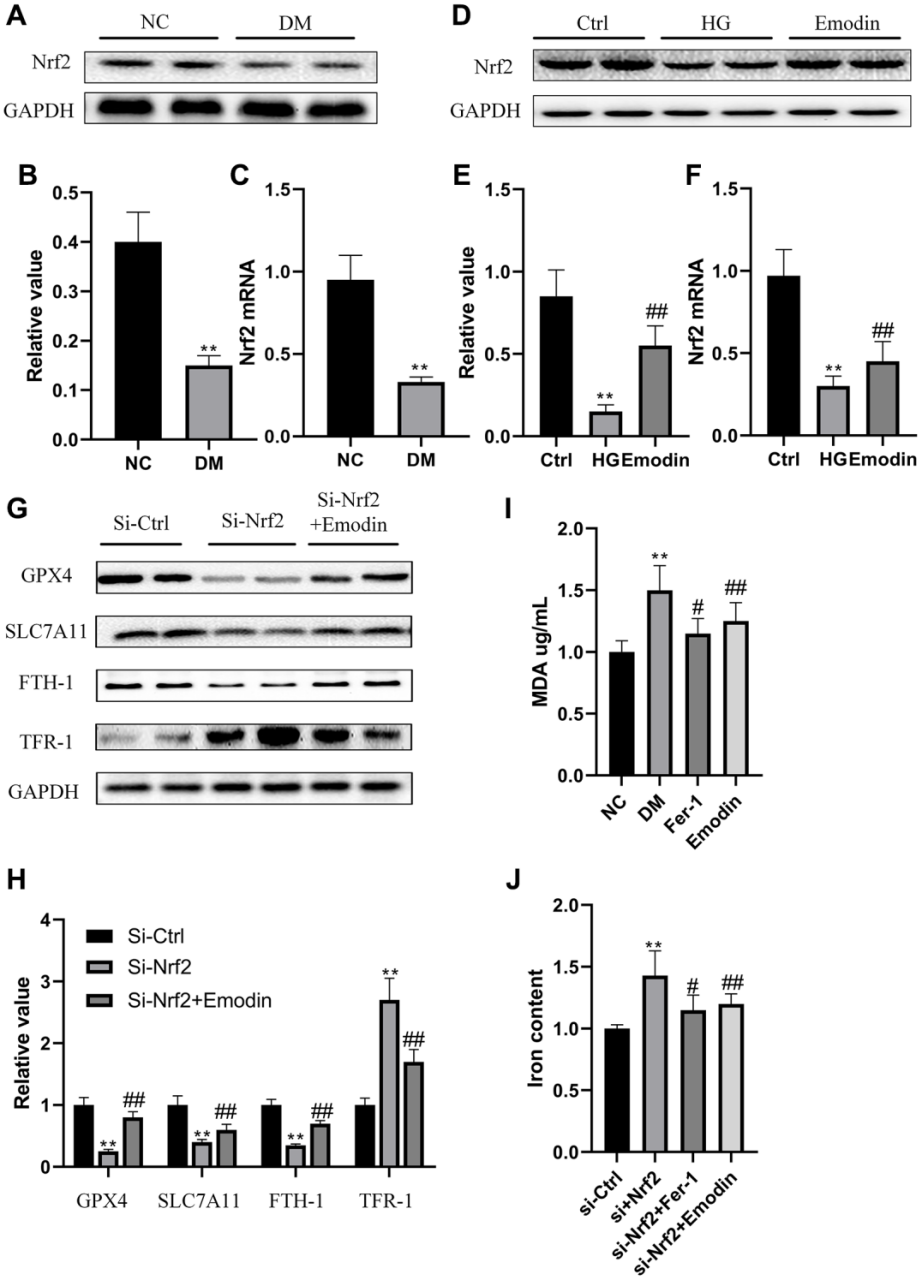
**The deletion of Nrf2 contributed to ferroptosis in HK-2 cells.** The Nrf2 protein expression was analyzed by western blotting and semi-quantitative analysis in renal tissues (**A**, **B**). The mRNA expression of Nrf2 (**C**). The expression level (**D**) as well as semi-quantitative analysis (**E**) of Nrf2 (**D**). The mRNA expression of Nrf2 (**F**). The expression levels as well as semi-quantitative analysis of ferroptosis related protein (**G**, **H**). The changes in iron and MDA (**I**, **J**) were measured. ^*^P < 0.05, ^**^P < 0.01 vs. normal group, ^#^*P* < 0.05, ^##^*P* < 0.01 vs. model group.

Nrf2 is a vital transcription factor that regulates antioxidant stress. Results gained from both western blotting and qRT-PCR indicated that Nrf2 expression was inhibited in DM group rats compared with ctrl group rats (Figure 4A–4C). *In vitro* experiment also revealed that there was a reduction in Nrf2 expression in HK-2 cells under the stimulation of 30 mmol/L glucose concentration for 72 h (Figure 4D–4F). In order to further investigate the functional role of Nrf2 in governing ferroptotic cell death. We deleted Nrf2 gene in HK-2 cells. Under the influence of high glucose medium, the absence of Nrf2 led to decreased expression of ferroptosis suppressor protein, including GPX4, SLC7A11and FTH-1 and elevated expression of TFR-1 (Figure 4G, 4H). The absence of Nrf2 also led to increased concentration of MDA (Figure 4I) and iron content (Figure 4J). Notably, the Nrf2 deletion mediated pathological changes were attenuated after Fer-1 intervention (Figure 4G–4J). These findings implied that the deletion of Nrf2 compromised the antioxidant stress ability and induced the occurrence of ferroptosis under high glucose condition.

### Emodin restored activation of Nrf2 pathway to alleviate ferroptosis

**Figure 5:**
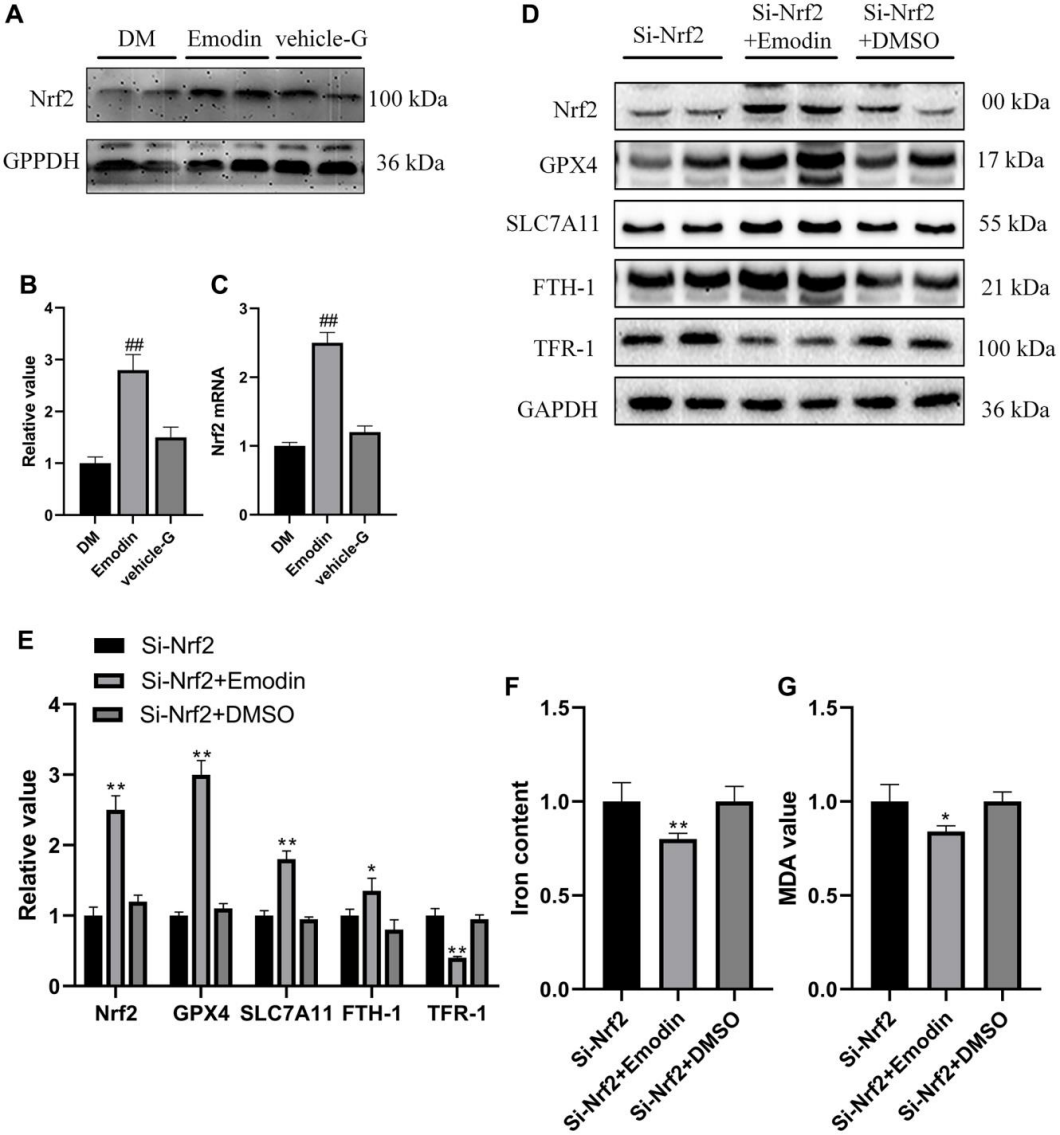
**Emodin inhibited the deletion of Nrf2 induced ferroptosis.** The expression level as well as semi-quantitative analysis of Nrf2 protein (**A**, **B**). The mRNA expression of Nrf2 (**C**). The expression levels as well as semi-quantitative analysis of ferroptosis indicators were measured in Si-Nrf2 cells with or without treatment (**D**, **E**). The changes in concentrations of iron and MDA were calculated (**F**, **G**). ^*^P < 0.05, ^**^P < 0.01 vs. Si-Nrf2 group, ^#^*P* < 0.05, ^##^*P* < 0.01 vs. DM group.

According to the results from Figure 5A–5D, it was indicated that expression levels of Nrf2 were significantly elevated in the Emodin treated diabetic rats compared with diabetic rats receiving no treatment. In order to further investigate the protective function of Emodin in treating DKD via promoting Nrf2 expression, we constructed Nrf2 Knock out cell model and Emodin intervention was employed. The results revealed that Si-Nrf2 group exhibited an elevated expression of Nrf2 after being treated with Emodin compared to that of the untreated Si-Nrf2 group (Figure 5D, 5E). Emodin intervention contributed to increased expression of ferroptosis suppressor proteins, including GPX4, SLC7A11and FTH-1 protein as well as decreased expression of TFR-1 in Si-Nrf2 group compared to that of untreated Si-Nrf2 group (Figure 5D, 5E). In addition, Emodin intervention also reduces the intensity of iron and MDA in the absence of Nrf2 (Figure 5F, 5G). Taken together, these above results implied that Emodin participated in the protection against high glucose induced ferroptosis via enhancing the expression of Nrf2.

### Emodin inhibited the RSL-3 induced cellular ferroptosis

**Figure 6:**
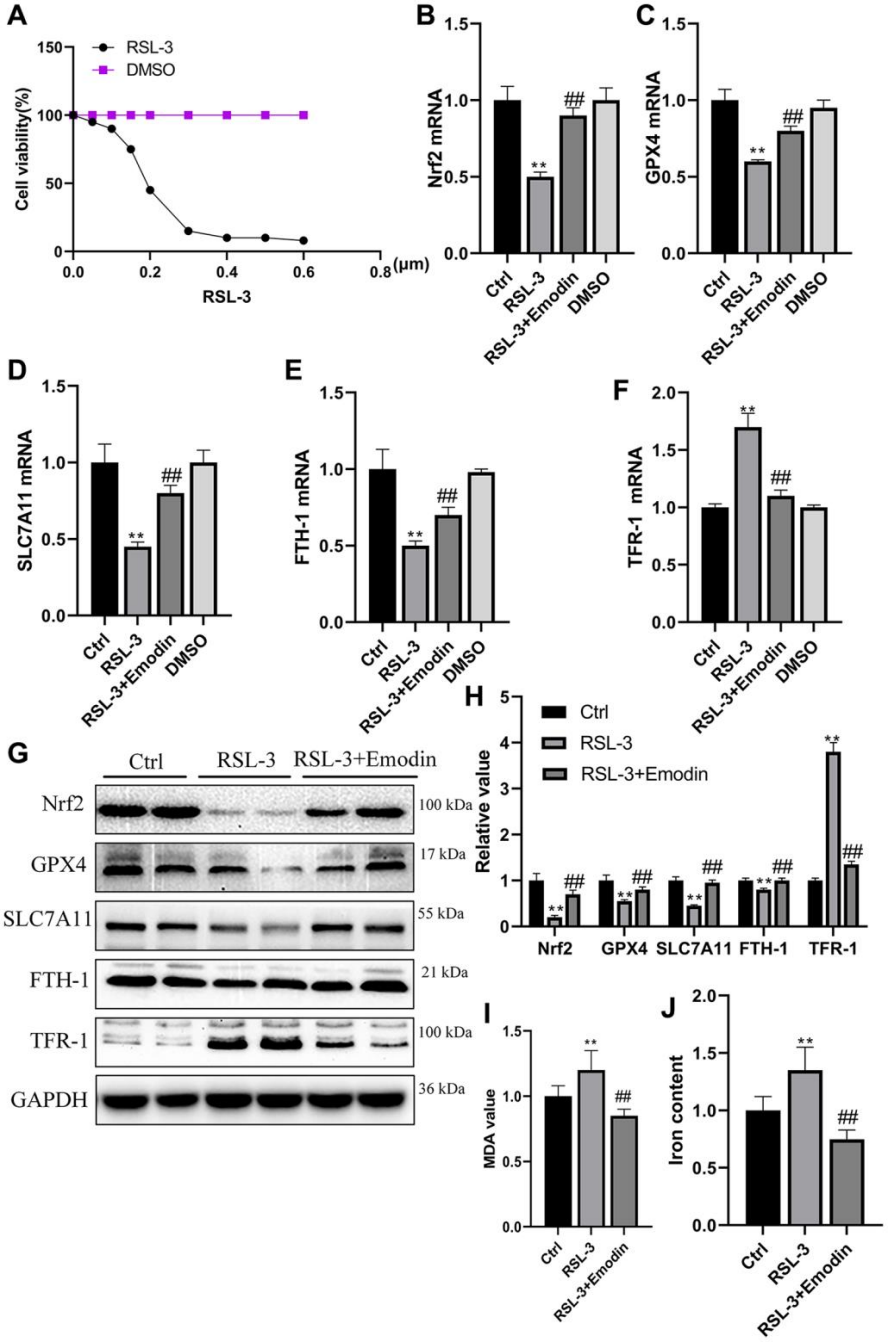
**The RSL-3 induced cellular ferroptosis was suppressed by the treatment of Emodin.** Different dose of RSL-3 was used to treat HK-2 cells exposed to culture medium containing 5.5 mmol/L glucose for 24 h (**A**). The mRNA expression of ferroptosis related indicators (**B**–**F**). The expression levels as well as semi-quantitative analysis of ferroptosis related proteins in RSL-3 group with or without Emodin treatment (**G**, **H**). The concentration of MDA and iron in RSL-3 group with or without Emodin treatment (**I**, **J**). ^*^P < 0.05, ^**^P < 0.01 vs. Ctrl group, ^#^*P* < 0.05, ^##^*P* < 0.01 vs. RSL-3 group.

The RSL-3 was an agent that used to induce cellular ferroptosis. With the increasing concentration of RSL, the HK-2 cells viability was significantly dropped in a dose dependent way (Figure 6A). Nrf2, GPX4, SLC7A11, FTH-1 and TFR-1 are generally employed as important hallmarks of ferroptosis. Their mRNA expression levels were found to be reduced in RSL-3 treated cells group, which were rescued after being treated with Emodin (Figure 6B–6F). The western blot results revealed that RSL-3 inhibited the expression of Nrf2, GPX4, SLC7A11 and FTH-1 accompanied the up-regulation of TFR-1 expression, which were significantly improved in Emodin treated cells (Figure 6G, 6H). The massive production of ROS and high iron accumulation induced by RSL-3 were reduced in Emodin intervention (Figure 6I, 6J). These results implied that Emodin exhibited an inhibitory effect on ferroptosis via increasing the expression of Nrf2.

### Emodin restored anti-oxidative stress system in DKD model

**Figure 7:**
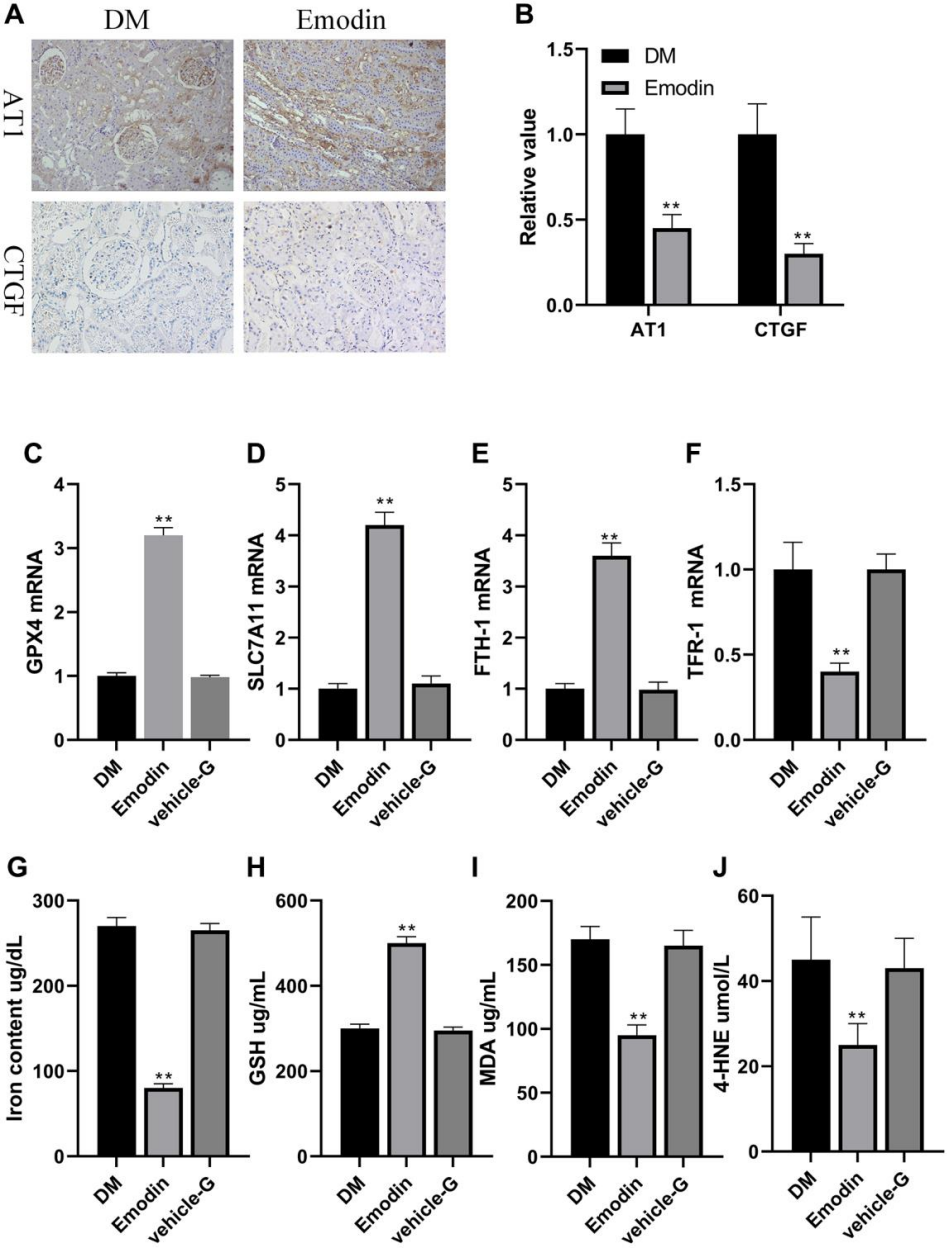
**Emodin attenuated ferroptosis related marker in rats with DN.** Immunohistochemical staining (**A**, 200× magnification) as well as semi-quantification (**B**) of AT1 and CTGF. The mRNA expression of ferroptosis related indicators in Emodin treated group (**C**–**F**). The concentration of iron content, GSH, MDA and 4-HNE (**G**–**J**). ^*^P < 0.05, ^**^P < 0.01 vs. model group.

The indicators regarding renal impairment in Emodin group were markedly attenuated in comparison to the DM group (Figure 7A, 7B). The mRNA expression of Nrf2, GPX4, SLC7A11 and FTH-1 were up regulated, and the mRNA expression of TFR-1 were reduced in Emodin treated group compared to the model group (Figure 7C–7F). Besides, we also found that Emodin reduced the iron concentration, and enhanced expression of GSH indicating that Emodin restored the capacity of anti-oxidation in kidney (Figure 7G, 7H). MDA and 4-HNE are the vital lipid peroxidation products participating in the occurrence of ferroptosis. The Emodin treatment significantly inhibited the generation of MDA and 4-HNE (Figure 7I, 7J). These results indicated that Emodin could inhibit ferroptosis by rescuing the impaired anti-oxidative stress capacity under diabetic ambience.

### Emodin ameliorated diabetic induced renal injury

**Figure 8:**
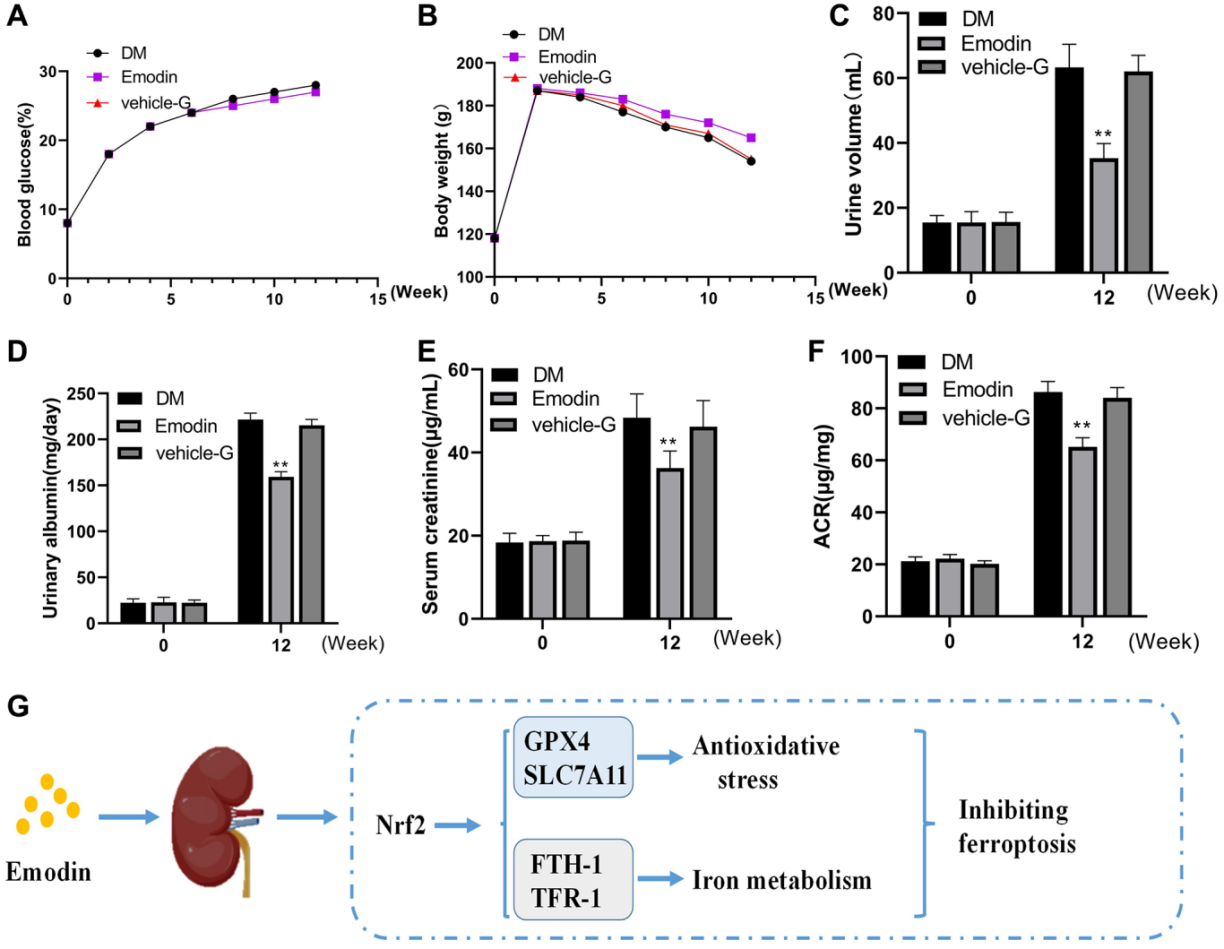
**Emodin treatment attenuated renal damage in STZ induced animal model.** The blood glucose and body weight were determined at different time (**A**, **B**). The urine volume, urinary albumin, serum creatinine and ACR were monitored at 0 and 12 weeks (**C**–**F**). The protective mechanism of Emodin against DKD through inhibiting ferroptosis via regulating Nrf2 pathway (**G**). ^*^p < 0.05, ^**^p < 0.01 vs. NC group, vs. DM group.

The changes in blood glucose and body weight exhibited no significant difference among the DKD group, Emodin group and vehicle-G group (Figure 8A, 8B). Emodin intervention attenuated the urine output in STZ induced diabetic rats. The mice in DKD group produced 60 mL volume of urine averagely, which reduced to a volume of 36 mL after Emodin treatment (Figure 8C). The levels of 24-h urinary albuminuria, serum creatinine and the ACRs were improved by Emodin treatment compared with untreated DKD group (Figure 8D–8F). The findings suggested that Emodin has the potential of attenuating the renal injuries in diabetic rats. And we also offer the schematic diagram of the protective action of Emodin against DKD related ferroptosis by enhancing the expression of Nrf2 in Figure 8G.

## DISCUSSION

Diabetic kidney disease (DKD) is a serious complication that often arises in individuals with diabetes [16]. The global prevalence of DKD is expected to reach 693 million by 2045 that makes it a top health issue to be dealt with urgently [17]. The population of the country is aging, and the elderly diabetes is gradually increasing. The incidence rate of people aged 55 to 64 years and over has reached 7%, and the incidence rate of people aged 70 years and over is even higher. More than 50% of elderly diabetes patients will have DKD, even renal failure, which has become one of the common diseases endangering the health and life of the elderly. Investigating the mechanism of DKD is a crucial step for creating a novel therapeutic option to prolong the life span of DKD patients. Mounting studies support the facts that ferroptosis serves a crucial mediator in the development of DKD, indicating that ferroptosis could be employed as a promising target for treatment of DKD [5].

Ferroptosis represents an emerging paradigm of cell death with distinctive attributes, including compromised antioxidant defenses, dysregulated iron homeostasis, and the agglomeration of lipid peroxidation products [18]. Ferroptosis has a negative effect on various diseases, which accelerated organ damage or aggravated disease progression, such as neoplasm, neurodegenerative disease and ischemic injury [19]. Accumulating studies have revealed that ferroptosis is closely associated with pathogenesis of DKD [8]. GPX4 holds a significant position as a renown antioxidant enzyme regulating ferroptosis defense system with is capacity of reducing phospholipid hydroperoxide [20]. The loss activity of GPX4 has been recognized as one of the biomarkers of ferroptotic cell death, and the specific GPX4 knock out or using ferroptosis agonist is contributed to renal tubular epithelial cells dying for ferroptosis.

However, the correlation between ferroptosis and DKD is still unclear. It is worth noticing that high expression of ACSL4, PTGS2, and NOX1, and decreased GPX4 and SLC7A11 levels were detected in patients with DKD [21]. Consistent with these findings, our *in vitro* and *in vivo* study confirmed that high glucose and erastin could both induce ferroptosis with the features of decreased GPX4 and SLC7A11 levels, and up-regulated levels of FTH-1, TFR-1, ROS, and MDA, which was rescued by treatment with Emodin. Collectively, these findings primarily reveal that GPX4 mediated defense system against ferroptosis was impaired by exposure to high glucose or the influence of diabetic condition, accompanied by the lower expression of GPX4, SLC7A11 and FTH-1, and elevated TFR-1 level, which could be regarded as biomarkers of ferroptosis. However, these adverse effects could be largely attenuated by treatment with Emodin suggesting Emodin improves the outcome of DKD mainly via inhibiting ferroptosis.

ROS accumulation not only acts a deteriorated role in the progression of DKD, but also is a typical pathological indicator of ferroptosis [22]. Kidney is capable of tolerating a small amount of ROS generation. But under the influence of diabetes, a persistent hyperglycemia condition induced massive amount of ROS production which exceeded the inside antioxidant system’s capacity of eliminating [23]. The accumulation of ROS damaged the structure of cell that facilitated the expression of pro-inflammatory factor, which led to the recruitment of macrophages and renal injuries [24]. The level of ROS was elevated in HK-2 cells exposing to high glucose medium. MDA and 4-HNE, the important biomarker of antioxidant system [25], were up-regulated under diabetic condition, which could be detected in both *in vivo* and *in vitro* experiments. Besides, the GPX4 function against ferroptosis was suspended by ROS. The above results implied that reducing ROS generation is an ideal way to inhibit ferroptosis that delay the progression of DKD.

Nrf2 serves as the primary transcription factor accountable for governing the function of antioxidant system to reduce intracellular ROS generation [26]. However, the persistent hyperglycemia condition reduced Nrf2 mediated antioxidant system’s resistance to eliminate ROS, which was strongly associated with the deterioration of DKD [27]. Considering the regulatory function of Nrf2 in mitigating oxidative stress, our study aimed to explore the potential of enhancing Nrf2 expression to attenuate DKD-associated ferroptosis, consequently delaying the progression of DKD. Consistent with the previous reports, the down regulation of Nrf2, GPX4 and SLC7A11 was observed in both *in vivo* and *in vitro* experiments under diabetic condition. The reduced expression of Nrf2, GPX4 and SLC7A11 leads to the weakened anti-oxidant capacity that failed to protect cells from oxidative stress, which finally led to the occurrence of ferroptosis.

Subsequently, we made the noteworthy observation that the administration of Emodin led to a notable enhancement in the expression of Nrf2, with the increased expression level of GPX4, SLC7A11 and FTH-1, and down regulated expression of TFR-1 was also observed. In order to provide additional validation regarding the inhibitory effect of Emodin on ferroptosis, we conducted RSL-3 induced ferroptotic cell death experiment, and found that the inhibition of Nrf2 expression under hyperglycemia condition was reversed by being treated with Emodin, thereby repairing anti-oxidant system of the kidney. The renal injuries of STZ-induced diabetic mice were also alleviated by using Emodin. Our findings shed a light on the renal protection of Emodin against DKD which is associated with inhibiting ferroptosis by enhancing Nrf2 expression.

The occurrence of albuminuria was observed in both DKD diagnosed and animal model with DKD, which recognized as a biomarker of the progression of renal injury [28]. Inhibition of albuminuria is beneficial in delaying the progression of DKD. Our study reveals that compared to normal group, albuminuria is detected in all STZ-induced diabetic rats. But the degree of albuminuria was less severe in STZ-induced diabetic rats treated with Emodin. In addition, the kidney function in diabetic mice was also improved after administration of Emodin. We confirmed that Emodin is capable of mitigating albuminuria induced by podocyte injury and improving renal function that led to improvement in DKD on the basis of results gained above.

In conclusion, our study highlights the pivotal role of ferroptosis in expediting the progression of DKD by impairing the antioxidant defense mechanisms. The treatment of Emodin exhibited its renal protective effect against DKD via inhibiting ferroptosis by up-regulating Nrf2. Collectively, the findings of this study offer a novel therapeutic option for the prevention and treatment of DKD.
